# Integrative structural modeling with small angle X-ray scattering profiles

**DOI:** 10.1186/1472-6807-12-17

**Published:** 2012-07-16

**Authors:** Dina Schneidman-Duhovny, Seung Joong Kim, Andrej Sali

**Affiliations:** 1 Department of Bioengineering and Therapeutic Sciences, Department of Pharmaceutical Chemistry, and California Institute for Quantitative Biosciences (QB3), University of California at San Francisco, San Francisco, USA; 2 UCSF MC 2552, Byers Hall at Mission Bay, Suite 503B, University of California at San Francisco, 1700 4th Street, San Francisco, CA, 94158, USA

**Keywords:** Small Angle X-ray Scattering (SAXS), Protein structure prediction, Macromolecular assembly, Integrative modeling

## Abstract

Recent technological advances enabled high-throughput collection of Small Angle X-ray Scattering (SAXS) profiles of biological macromolecules. Thus, computational methods for integrating SAXS profiles into structural modeling are needed more than ever. Here, we review specifically the use of SAXS profiles for the structural modeling of proteins, nucleic acids, and their complexes. First, the approaches for computing theoretical SAXS profiles from structures are presented. Second, computational methods for predicting protein structures, dynamics of proteins in solution, and assembly structures are covered. Third, we discuss the use of SAXS profiles in integrative structure modeling approaches that depend simultaneously on several data types.

## Introduction

SAXS is becoming a widely used technique for low-resolution structural characterization of macromolecules in solution [1-5]. The major advantage of SAXS compared to other structural characterization techniques is that it can be performed under a wide variety of solution conditions, including near physiological conditions, and for a wide range of molecular sizes. The experiment is typically performed with ~0.5–10.0 mg/mL of a macromolecular sample in a ~15–30 μL volume, and usually takes less than a few minutes on a well-equipped synchrotron beam line. In addition, recent technological advances allow in-house laboratory data collection without a beam line X-ray source.

The SAXS experiment results in a small-angle X-ray scattering intensity of a sample (a macromolecule in a buffer) as a function of spatial frequency [6-8]. The SAXS profile of the macromolecule is then produced by subtracting the SAXS profile of the buffer from the SAXS profile of the sample. Because of rotational averaging, the information content of a SAXS profile is significantly lower compared to that of a diffraction pattern in X-ray crystallography or even a density map from electron microscopy (EM). Nevertheless, SAXS can provide shape information about proteins and macromolecular assemblies that are not amenable to X-ray crystallography, NMR spectroscopy, or EM. Information about the radius of gyration, volume, and mass can be easily extracted from the profile [8,9]. The profile can be converted into an approximate distribution of pairwise electron distances in the macromolecule (i.e. the pair-distribution function) *via* a Fourier transform [7,8,10]. Moreover, SAXS can be used to study the dynamics of the system [11,12], not only a static structure.

SAXS profiles can be used for computation of so called *ab initio* 3D shapes that are consistent with the measured pair-distribution function [13-15]. SAXS profiles can also be used for atomic resolution modeling in multiple modeling applications, including determination of biologically relevant states from the crystal [16], binding of small molecules [17], comparative protein structure modeling [18], fold recognition [19-21], protein domain assembly [22], assembly of protein complexes [23-25], modeling of a perturbed conformation (*eg*, modeling active conformation starting from non-active conformation) [26], and modeling of an ensemble of conformations that represent solution conformational ensemble [27-29]. Moreover, SAXS profiles can provide structural information about oligomeric states and interactions between proteins [30,31].

Characterizing structures of multi-subunit complexes generally benefits from using varied experimental datasets [32,33]. In this hybrid or integrative approach, the datasets are encoded into a scoring function used to evaluate candidate models generated by a sampling method. Due to the simplicity of data collection, SAXS profiles are attractive candidates for combination with other computational and experimental techniques by the integrative approach [24].

In this review, we describe the recently developed computational methods that were inspired by the ability to collect high-accuracy SAXS profiles. First, the methods for computing theoretical SAXS profiles from structures are presented. Second, we describe how SAXS is incorporated into computational methods for predicting protein structures, dynamics of proteins in solution, and assembly structures. Finally, we describe the integrative modeling structure approach that depends simultaneously on several data types and suggest data types that are complementary to SAXS.

## Computing the theoretical scattering

Computation of an accurate theoretical SAXS profile from an atomic model is critical for including SAXS data in any modeling application. Progress was made based on the recent availability of high resolution SAXS datasets [1-3,5]. Theoretical SAXS profile calculation from the coordinates of atomic models requires spherical averaging because of random orientations of macromolecules in solution. Since the observed scattering profile is the difference between the scattering of the target macromolecule with its ordered hydration layer and the excluded volume that takes into account the missing scattering of bulk solvent, methods for calculating SAXS profiles have to account for the excluded volume of bulk solvent and the hydration layer. As a result, the approaches for profile computation generally differ in the methods used for spherical averaging, treatment of the excluded volume, and treatment of the hydration layer (Table 1).

**Table 1:** Methods for theoretical profile calculation

**Method**	**Spherical Averaging**	**Hydration Layer**	**Representation**	**Availability**
CRYSOL [34]	Multipole expansion	Implicit water layer based on envelope function	Atomic	Server, download http://www.embl-hamburg.de/biosaxs/crysol.html
solX [35]	Debye formula	-	Atomic	
ORNL_SAS [36]	Monte-Carlo sampling	Implicit water layer	Grid representation	Download http://www.ornl.gov/sci/csd/Research_areas/MS_csmb_comp_methods.htm
SoftWAXS [37]	Numerical quadrature	Implicit water layer	Atomic	
Fast-SAXS [38]	Debye formula	Explicit placement of water molecules	Coarse-grained residue level	http://yanglab.case.edu/software.html
Park et al. [39]	Spherical quadrature	Explicit placement of water molecules	Atomic	
Stovgaard et al. [40]	Debye formula	-	Coarse-grained, 1 or 2 points per-residue	
AXES [41]	Numerical quadrature	Explicit placement of water molecules	Atomic	Server http://spin.niddk.nih.gov/bax/nmrserver/saxs1/
FoXS [42]	Debye formula	Implicit water layer based on surface accessibility	Atomic or coarse-grained residue level	Source code, server, download, Chimera http://salilab.org/foxs/
AquaSAXS [43]	Cubature formula	AquaSol solvent density map	Atomic	Server http://lorentz.dynstr.pasteur.fr/aquasaxs/aquasaxs_submission.php
Virtanen et al. [44]	Debye formula or Cube model	HyPred based on MD simulations	Atomic, MD simulation	
Zernike Polynomials [45]	Zernike polynomial expansions	Hydration layer from voxelized representation	Atomic	Source code, server, download http://sastbx.als.lbl.gov/cgi-bin/intensity.html

Spherical averaging methods need to balance accuracy and run-time performance. Spherical averaging can be computed directly from all pairwise interatomic distances using the Debye formula [24,35,42,46]. CRYSOL [34] uses multipole expansion for fast calculation of a spherically averaged scattering profile. Other options include Monte-Carlo sampling [36], numerical quadrature [37,41], cubature formula [43], and Zernike polynomial expansions [45]. Coarse graining that combines several atoms in a single scattering center can also be used to speed up the calculation [38,40].

The excluded volume term typically depends on the shape of the molecule by calculating the scattering assuming an electron density equivalent to the bulk solvent [34,47,48]. Alternatively, it is possible to represent the excluded volume by explicit placement of water molecules [41]. However, accurate approximation of the excluded volume is challenging because the total volume varies significantly depending on a set of values of atomic radii. Therefore, some methods allow adjustment of the excluded volume of the molecule for optimal fitting to the experimental SAXS profile [34,42,43].

The hydration layer can be treated explicitly by introducing water molecules [38,39,41] or using pre-computed solvent density maps [43,44]. Implicit hydration layer models surround the molecule with a continuous envelope of adjustable density [34,36,42].

There is generally a trade-off between the accuracy and speed of computation. For example, if a method is used to evaluate a profile fit for multiple models, it has to be fast compared to a method that will be used to compare a single structure to the SAXS profile. Wide angle scattering requires more accurate methods to account for atomic resolution details that can be seen at wide angles [39,41,44].

The theoretical profile is typically fitted to the experimental one by minimization of the *χ* value [34]:

(1) $$\chi =\sqrt{\frac{1}{M}\;\sum_{i=1}^{M}{\left(\frac{I_{\exp}\;\left(q_{i}\right)-cI\;\left(q_{i}\right)}{\sigma \left(q_{i}\right)}\right)}^{2}}$$

where *I*_*exp*_*(q)* and *I(q)* are the experimental and computed profiles, respectively, *σ(q)* is the experimental error of the measured profile, *M* is the number of points in the profile, and *c* is the scaling factor. Sometimes, there are additional fitting parameters that require optimization during fitting, such as the excluded volume of the protein, the density of the hydration layer [34,42,43], and buffer rescaling factor [41]. The major problem with *χ* is that its values are comparable only for the same experimental profile since it depends on the profile experimental error. Therefore, one can compare the fitting quality between two models against the same profile using *χ,* but cannot compare the fit of one model against two different experimental profiles.

**Figure 1:**
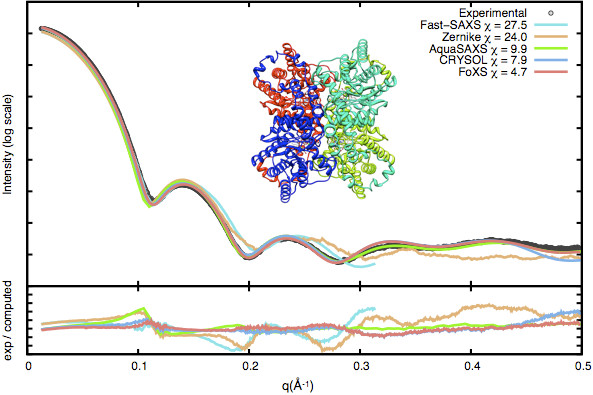
Theoretical SAXS profiles of glucose isomerase from five programs fitted against the experimental SAXS profile.

To assess the performance of different profile calculation programs (Zernike polynomials, Fast-SAXS, AquaSAXS, CRYSOL and FoXS), we compute the theoretical scattering for a model protein glucose isomerase [PDB:2G4J] (Figure 1) and fit it to the experimental profile. A high accuracy SAXS profile (*q*_*max*_ = 0.5 Å^-1^) was collected and analyzed at the Advanced Light Source SIBYLS beam line (BL12.3.1), as described previously [1]. The molecule includes approximately 12,000 atoms. FoXS and CRYSOL provide the most accurate fit with *χ* values of 4.7 and 7.9, respectively, in less than 8 seconds.

## Protein structure prediction

### X-ray structure vs. solution structure

**Figure 2:**
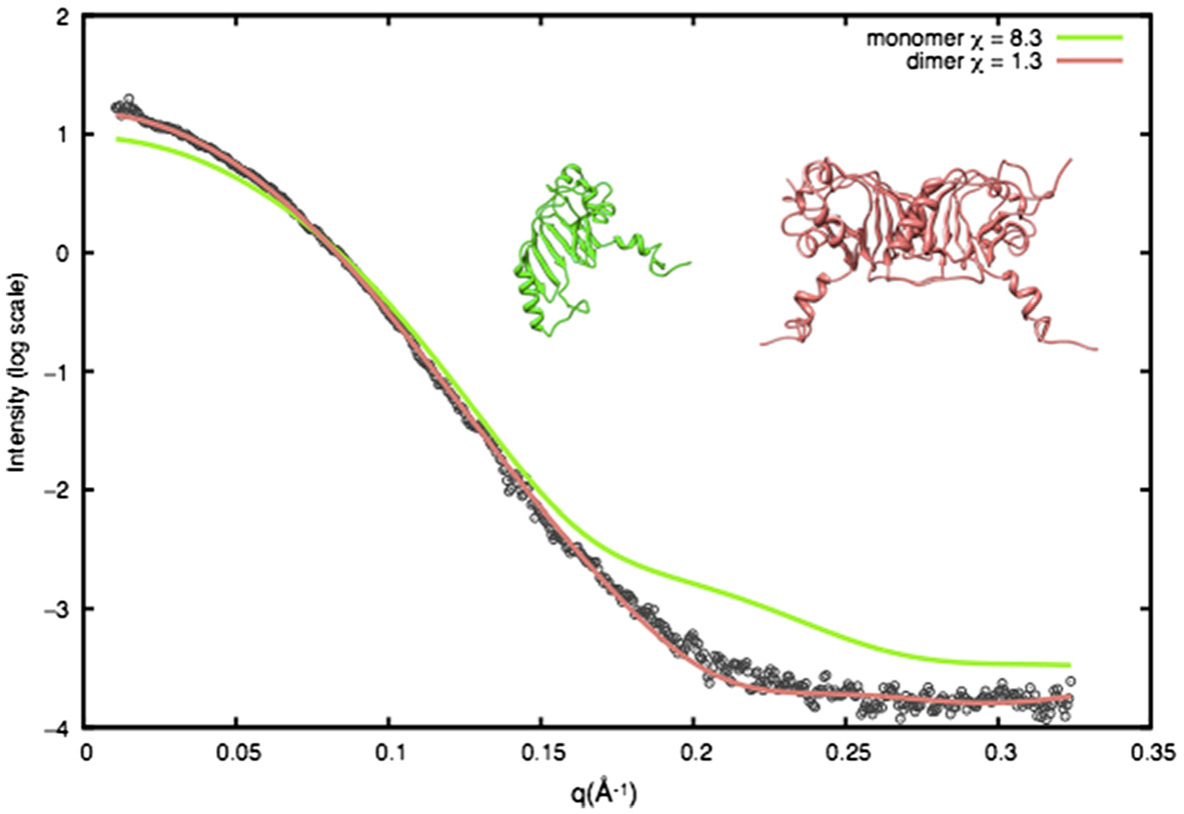
**Yeast Nup145N (443–605) crystal structure [PDB:3kep] monomer (green) and dimer (red)*versus*the solution SAXS profile (black).**

The most straightforward application of SAXS is for comparing a crystallographic structure with a solution structure. The theoretical profile computed from the X-ray structure is compared with the experimental one. The comparison can help in identifying biologically relevant oligomeric states or the solution quaternary structure of the protein. For example, a comparison of the SAXS profile of yeast Nup145N (443–605) with the crystal structure [PDB:3KEP] confirmed the existence of a dimer seen in the asymmetric unit in solution [49] (Figure 2).

**Figure 3:**
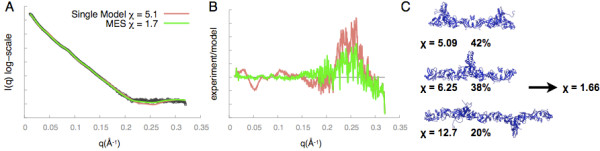
**FoXS-MES server output for ensemble fit (green) of the SAXS profile (black)*versus*a single conformation fit (red) for XLF-XRCC4 filaments. A**) Fit plot, **B**) Residuals plot, and **C**) Ensemble structures and weights.

A system can exist in a mixture of several states in solution, such as monomer-dimer equilibrium. In such cases, fitting of a weighted average of theoretical profiles can be attempted for comparison with the experimental profile. The program OLIGOMER implements a non-negative linear least-squares algorithm to find the weights of the theoretical profiles that minimize the discrepancy with the experimental profile [50]. FoXS webserver allows fitting of up to five weighted computed profiles to the experimental profile. The selection of weighted profiles is performed using Minimal Ensemble Search (MES) that is based on a genetic algorithm [28]. FoXS-MES webserver was previously used to determine the length and composition of the XLF-XRCC4 filaments in solution [51]. The XLF-XRCC4 complex forms filaments in the crystal lattice [51]. Theoretical scattering profiles were computed for various filament lengths with FoXS, followed by MES [28] that selects a combination of various filament lengths to optimize the fit to the experimental SAXS profile. The best fit obtained with the single filament had χ value of 5.09, while the minimal ensemble of three different filaments reduced the χ value to 1.66 (Figure 3).

### Comparative modeling and missing fragment modeling

**Figure 4:**
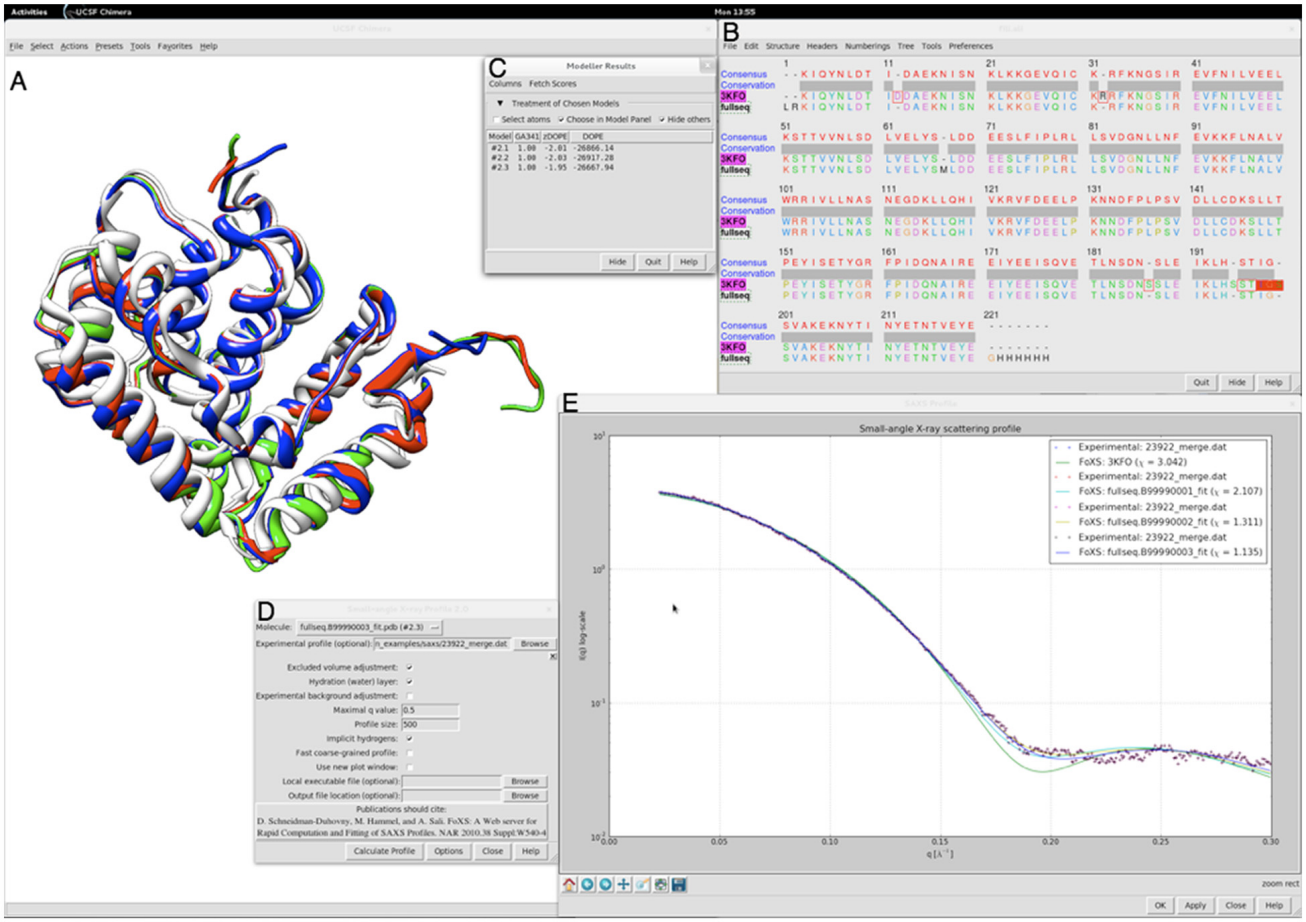
**Comparative modeling and SAXS profile fitting using the Chimera visualization package.** MODELLER was used to add missing residues and a His tag on the C-terminal domain of Nup133 (see sequence alignment in the upper right window). Three models were generated (red, green and blue) using a template structure [PDB:3KFO] (white). The FoXS interface (lower left window) was used to fit the profiles computed for the X-ray structure and the models to the experimental SAXS profile (lower right window). **A**) model window, **B**) sequence alignment window, **C**) model scores, **D**) FoXS interface window, and **E**) FoXS output window.

If X-ray structures have unresolved regions, such as side-chains, loops or His tags that are not seen in the electron density, modeling of a complete structure based on the sequence used for SAXS data collection is critical for a useful comparison of computed and experimental profiles. The fraction of missing atoms in a structural model results in an almost double fraction of missing distances in the calculation of the theoretical profile. For example, if 5% of residues are missing from the X-ray structure of 100 residue protein, the fraction of missing distances in computation of the theoretical profile is almost 10%. If only a homologous structure of the studied protein is available, it is also important to model the target sequence by comparative modeling [18,52], since even at high sequence identity the homologous proteins may have variable loop lengths. In addition, an experimental SAXS profile can help in distinguishing between alternative structural models [41]. Structural models can be generated using a variety of programs and web services, such as HHpred [53] (http://toolkit.tuebingen.mpg.de/hhpred), M4T [54] (http://manaslu.aecom.yu.edu/M4T/), SWISS-MODEL [55](http://swissmodel.expasy.org/), Robetta [56] (http://robetta.bakerlab.org/), I-TASSER [57] (http://zhanglab.ccmb.med.umich.edu/I-TASSER/), and ModWeb [58] (http://salilab.org/modweb). Both, the modeling task and profile calculation can be performed using UCSF Chimera molecular graphics program [59] that has an interface to MODELLER [18] for automated comparative modeling and FoXS [42] for profile calculation and fitting. For example, the structure of the C-terminal domain of Nup133 was characterized by both X-ray crystallography and SAXS. Using Chimera, the X-ray structure [PDB:3KFO] is fitted to the SAXS profile with the resulting *χ* value of 3.04 (Figure 4). After adding the missing residues and His tag with MODELLER using the Chimera interface, the fit improves significantly (*χ =* 1.1*)*, especially for 0.17 Å^-1^ < *q <* 0.22 Å^-1^ (Figure 4).

### Fold and shape recognition

A SAXS profile can be utilized in structural modeling of protein sequences for which template structures cannot be identified using a sequence similarity search. Zheng and Doniach [20] used a SAXS profile to filter structures generated by gapless threading on the templates. The recently developed SAXSTER method [21] integrates a SAXS-based scoring function with the MUSTER threading algorithm. The SAXS profile fit score is combined with the threading alignment score, resulting in a higher accuracy model compared to that from MUSTER without using a SAXS profile.

If comparative modeling or fold recognition methods fail to produce accurate models, it is possible to search for proteins with similar overall folds or shapes using a SAXS profile of a given protein. The DaRa server [60] searches for similarity among the theoretical scattering profiles pre-computed from thousands of PDB structures. The Shape Search Engine in SASTBX package (http://sastbx.als.lbl.gov/cgi-bin/shapeup.html) represents the structures using Zernike polynomials and performs a rapid shape comparison against PDB [61], PISA [62], and 3Dcomplex databases [63].

## Dynamics modeling

SAXS is a valuable tool in characterizing the ensemble of conformations sampled by a macromolecule in solution. Depending on the variance of the solution ensemble, alternative conformations or an ensemble of conformations may be needed to address the discrepancy between an experimental SAXS profile and an X-ray structure. For example, the protein can be in a non-active conformation in the crystal and in the active conformation in solution. In such a case, one needs to model alternative conformations starting with the X-ray structure. Multi-domain proteins are likely to be conformationally variable in solution, in part depending on the length and composition of the segments between domains. Accurate profile fitting in this case requires a set of multiple conformations.

### Fitting a single perturbed conformation to a SAXS profile

One possible approach is to use a SAXS profile as a filter for previously generated conformations: thousands of conformations are generated first and the scattering profile of each conformation is computed and fitted to the experimental profile. There are several ways to generate alternative conformations. The BUNCH method [23] uses simulated annealing approach where the domains are kept rigid and linkers are flexible chains composed of dummy residues. The BILBOMD method [28] uses multiple time-step Molecular Dynamics at high temperatures while keeping the domains rigid and the linkers flexible.

Alternatively, it is possible to use a SAXS profile directly in the optimization. Monte Carlo based method can be used for sampling relative domain orientations [24], where the gradient of *χ*^2^ is used for guiding the optimization. In a different approach, Normal Mode Analysis has been utilized for fitting the pair distribution function derived from a SAXS profile [64]. A recent method by Zheng and Tekpinar [65] uses a coarse-grained Elastic Network Model (1 bead per residue) coupled with coarse-grained SAXS profile calculation that includes an implicit hydration layer.

Even if a good fit of a SAXS profile can be obtained with a single conformation, it is still possible that the protein is flexible in solution [11]. Kratky [6] and Porod-Debye [9,29] plots should be used to distinguish between rigid and flexible proteins. However, this classification can be difficult for some proteins, such as those with rigid domains with long flexible loops.

### Modeling an ensemble of solution conformations

If the SAXS profile indicates that a protein is flexible in solution [9], we can attempt to fit the profile with an ensemble of conformations. Selection of a representative ensemble out of thousands of conformations is challenging since the ensemble size is not known and the number of possible ensembles is enormous. Several approaches exist to select an ensemble that fits a SAXS profile from a pool of multiple conformations. EOM [27] and BILBOMD Minimal Ensemble Search (MES) [28] use a genetic algorithm for ensemble selection. In the BSS-SAXS approach [66], the conformations are first clustered by RMSD and profile similarity into a small number of clusters, followed by a Bayesian-based Monte-Carlo method to optimize the weights of each cluster. Similarly, the EROS method [67] samples the conformations with a replica exchange Monte Carlo method, clusters the models, and optimizes cluster weights. Typically, significant improvement in the profile fit can be seen with a small ensemble size of 2 to 5 weighted conformations in MES or 10 conformations in EOM. In practice, the solution ensemble size might be much larger, but the selected ensemble is sufficient to fit the experimental profile and should be viewed as a minimal ensemble to explain the data. Measures, such as RMSD, NSD, radius of gyration, and maximal diameter are typically used to assess the ensemble variance [27,28]. If the variance in these parameters in the selected ensemble is as large as in the initial pool of conformations, the protein is likely to be flexible. Alternatively, macromolecules with distinct values of RMSD, radius of gyration, and maximal diameter, as compared to the initial pool, are less flexible and may have a limited number of conformers in solution.

**Figure 5:**
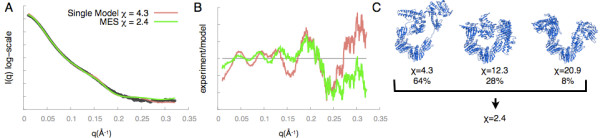
**FoXS-MES ensemble fit (green) of the SAXS profile (black)*versus*a single conformation fit (red) for Mre11-Rad50. A**) Fit plot, **B**) Residuals plot, and **C**) Ensemble structures and weights.

SAXS was used to study the flexibility of the Mre11-Rad50 dimers in solution with and without the ATP ligand [68]. The SAXS profiles show a transition from a flexible to ordered conformation upon ATP binding. The BILBOMD method [28] was used to model the solution ensemble of the Mre11-Rad50 dimers [68]. Multiple models were generated by Molecular Dynamics, followed by fitting to a SAXS profile with FoXS and MES. While the best fitting model had a χ value of 4.3, the minimal ensemble of three different models reduced the χ value to 2.9 (Figure 5).

### Time-resolved SAXS

Recent advances in time-resolved SAXS experiments provide a unique opportunity for data collection on with a ~10 microsecond resolution [69]. Structural information on the unfolded ensemble and early folding intermediates of proteins can be obtained by application of continuous or stopped-flow mixers [70-72]. In principle, this information can be used for validation of folding trajectories [69] generated by all atom Molecular Dynamics simulations that can cover similar timescales [73]. Processes such as virus shell assembly [74], virus maturation [75,76], and amyloid formation [77] can also be monitored by time-resolved SAXS. The minimal number of independent components (conformations or oligomeric states) in a set of time-resolved SAXS profiles can be inferred from singular value decomposition (SVD) analysis [7,30,50,78-80]. The method gives the minimal number of eigenvectors that account for the set of time-resolved profiles. The approach provides a lower bound for the actual number of components in the system.

## Assembly modeling

### Template-based modeling

Similarly to the modeling of individual proteins, it may be possible to model an assembly using standard comparative modeling techniques. The approach requires structural templates for the sequences of the target assembly that cover the entire assembly or a sufficiently overlapping set of its subunits. A comparative model of an assembly can be assessed by comparison to the experimental SAXS profile, as well as additional scoring functions that score subunit interaction interfaces [24]. Recently, several approaches for template-based pairwise protein docking were developed, including KBDOCK [81] (http://kbdock.loria.fr/), GWIDD [82,83] (http://gwidd.bioinformatics.ku.edu), 3DID [84] (http://3did.irbbarcelona.org/), SCOPPI [85] (http://www.scoppi.org/), and PRISM [86].

### Protein-protein docking

If atomic structures of subunits in a protein-protein complex are available, computational pairwise docking methods can be applied to predict the complex structure. The accuracy of computational protein-protein docking remains unsatisfactory despite recent advances in the docking methods [87,88]. Combining computational docking with SAXS is a promising approach towards increasing the accuracy of computational docking. There are several methods for rigid docking with a SAXS profile. DIMFOM and GLOBSYMM [23] are based on the CRYSOL program for SAXS profile fitting with a simplified sampling algorithm, where the structure of one monomer is rolled over the surface of the other; however, no interface optimization is performed. SASREF [23] uses Simulated Annealing for orientation sampling and CRYSOL for fit evaluation. In another method, the scoring function combines SAXS and interface complementarity terms, with orientation sampling by a local search method that requires a relatively accurate initial configuration [24]; in the absence of the initial configuration, the method starts from 1000 random orientations. Two recently developed methods, pyDockSAXS [89] and FoXSDock [90], integrate global search docking programs, energy based scoring functions, and a SAXS fit score. pyDockSAXS uses FTDock [91] for sampling complex orientations, pyDock [92] for energy-based scoring, and CRYSOL for SAXS scoring. FoXSDock uses PatchDock [93] for orientation sampling, FireDock [94] for refinement and energy-based scoring, and FoXS for SAXS scoring. Both methods nearly double the success rate relative to docking alone: pyDockSAXS succeds to rank a near native model within top 10 predictions for 43% of the Benchmark 2.0 cases (14 of the 84 cases were excluded because of incomplete unbound or bound structures), and FoXSDock succeeds in 60% of cases on the same subset of Benchmark 2.0 cases, compared to less than 30% for docking alone and 23% for SASREF that uses only SAXS score without interface optimization. The increase in the success rate of FoXSDock compared to pyDockSAXS is most likely due to the increased resolution of configurational sampling.

**Figure 6:**
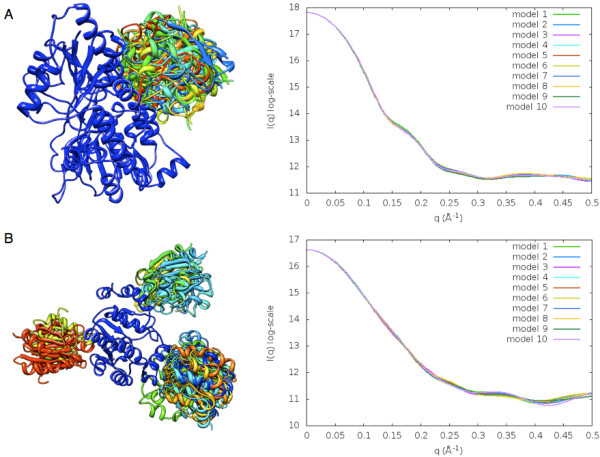
**Docking models with similar shapes generate similar profiles. A**) Top 10 docking models for adrenodoxin reductase-adrenodoxin complex [PDB:1E6E], and **B**) top 20 docking models for PAPS reductase-thioredoxin complex [PDB:2O8V].

The ability of a SAXS profile to distinguish between candidate docking models depends on the shapes of input proteins. For example, if one of the docked proteins has a globular shape, all the complexes with the correct binding site on the other protein will have similar shapes, almost identical SAXS profiles, and a similar range of low χ values, irrespective of the ligand orientation (Figure 6a). If the shape of one of the proteins is symmetric, there will be a number of clusters with similar shapes, SAXS profiles, and low χ values (*eg*, three clusters for the triangular receptor shape; Figure 6b).

When transient protein-protein interactions are modeled utilizing a SAXS profile, the solution sample may contain a mixture of monomers and complexes. The modeling procedure has to fit the experimental profile including the monomeric and complex models, and the weights of each component have to be determined. The option to account for such polydispersity was recently added to SASREF [95].

### Assembly of multi domain proteins

Multi-domain proteins can be modeled from single domain structures using a SAXS profile of the whole protein. Once the linkers between the domains are added to obtain an initial full-length structure, it is possible to refine the initial model to match the SAXS profile as well as possible. For short linkers, where significant contact between the domains is expected, protein-protein docking with distance constraints to connect the domains can be used. The BUNCH program [23] is designed specifically for the multi-domain assembly task, where the domains are represented by rigid bodies and the linkers are represented by a point per residue. Simulated annealing is used to optimize the domain positions and linker conformations. Additionally, BUNCH can simultaneously fit additional profiles that correspond to domain deletion mutants.

### Macromolecular assembly

Modeling of multi-subunit complexes based only on a SAXS profile of the complex is a challenging task, since ambiguous results are possible even for only two subunits (Figure 6). It is important to compute and analyze the set of all models consistent with the data, since multiple configurations can often have comparably good fits. Moreover, sampling of complex configurations is difficult, since each subunit adds six additional degrees of freedom (three rotational and three translational parameters) to the optimization problem. SASREF [23] can assemble multiple subunits using Simulated Annealing. It can simultaneously fit multiple SAXS profiles from the sub-complexes, reducing the ambiguity of the fit.

The assembly problem is further complicated by missing disordered fragments whose electron density needs to be accounted for in SAXS profile fitting. The recently developed CORAL method [95] addresses this problem by combining SASREF and BUNCH. In CORAL, distance restraints are added between the endpoints of consecutive protein domains for the sampling by Simulated Annealing. For each generated configuration, low-resolution linkers (a point per residue) are added using the RANLOGS library [95] for the calculation of a theoretical SAXS profile and the corresponding fit score.

Modeling of symmetric assemblies with cyclic (*C*_*n*_) or dihedral (*D*_*n*_) symmetry starting from a monomeric structure is possible with GLOBSYMM [23] that performs a brute-force search of symmetric configurations. In an integrative approach that combines stereo-chemical restraints, an atomic distance-dependent statistical potential, and a SAXS score, a symmetry term was added to the scoring function to assemble the homo-tetramer of D-xylose isomerase [24]. The FoXSDock method [90] can also be applied by replacing the pairwise global search module with SymmDock [96,97].

## Integrative modeling with SAXS profiles

Due to the difficulty of determining the atomic structures of multi-subunit complexes by X-ray crystallography and NMR spectroscopy, structural characterization of these assemblies generally benefits from using varied experimental datasets. This hybrid or integrative approach involves a computational encoding of the standard scientific cycle of gathering data, proposing hypotheses, and then gathering more data to test and refine those hypotheses [32,33]. First, the information is gathered from experimental data, statistical tendencies such as atomic statistical potentials, and physical laws such as molecular mechanics force fields, and converted into a score that assesses a structural model. Second, the resolutions of the representation and the corresponding scoring function for model evaluation are selected. The resolution of the representation depends on the quantity and resolution of the available information and should be commensurate with the precision of the final ensemble of good scoring models (when a single state is determined): different parts of a model may be represented at different resolutions, and one part of the model may be represented at several different resolutions simultaneously. The scoring function evaluates whether or not a given model is consistent with the input information, taking into account the uncertainty in the information. Third, the search for models that score well is performed using any of a variety of sampling and optimization schemes. Fourth, the ensemble of good-scoring models needs to be clustered and analyzed to ascertain their precision and accuracy, and to check for inconsistent information. Analysis can also suggest what are likely to be the most informative experiments to perform in the next iteration. Integrative modeling iterates through these stages until a satisfactory model is built.

SAXS data can be easily used as part of the integrative modeling. The first stage of data collection is rapid and simple with a sufficient amount of purified sample. Moreover, it is possible to collect data for sub-complexes and then use several profiles in modeling. Multiple methods exist for scoring a given model, given a SAXS profile, both for atomic and coarse-grained representations. The open source Integrated Modeling Platform (IMP) software suite [33,98] includes support for a SAXS score based on the FoXS method for models at the atomic and coarse-grained (one point per residue) resolutions.

Since a SAXS profile provides information about the global shape of a complex, the most informative complementary datasets are related to the interface composition. Information about interface residues can come from a variety of experiments, such as NMR chemical shift perturbations (CSPs) or saturation transfer (SAT) experiments [99,100], mutational analysis, hydrogen/deuterium exchange mass spectrometry (H/DX-MS) [101], and computational interface prediction methods [102]. Data from NMR residual dipolar couplings (RDCs) [103-105] and rotational diffusion tensor [106,107] resolve the relative orientation of two molecules, while a SAXS profile can help to determine the relative translation. Distance restraints from NMR [108], cross-linking/mass spectrometry [109] and FRET experiments can be easily converted to additional modeling restraints [110]. While additional datasets can be used together with SAXS to guide the modeling, it is also possible to use them for validation of models obtained from modeling with SAXS data only.

## Conclusions

Measurement by advanced instrumentation leads to more accurate SAXS profiles, requiring advanced computational methods for data interpretation. SAXS profiles are being incorporated into multiple modeling tasks, including single protein structure prediction, macromolecular assemblies modeling, characterization of flexible systems, as well as modeling of dynamics. Nevertheless, ambiguous modeling results are possible because a SAXS profile is spherically averaged at limited resolution. Thus, different models may have similar SAXS profiles all of which are consistent with the experimental profile. The integrative modeling approach that combines multiple datasets may help in discriminating among these ambiguous models.

## Authors’ contributions

D.S. wrote the review jointly with SJ. K. and A.S. All authors read and approved the final manuscript.
